# Live-Attenuated *Salmonella*-Based Oral Vaccine Candidates Expressing PCV2d Cap and Rep by Novel Expression Plasmids as a Vaccination Strategy for Mucosal and Systemic Immune Responses against PCV2d

**DOI:** 10.3390/vaccines11121777

**Published:** 2023-11-28

**Authors:** Khristine Kaith Sison Lloren, John Hwa Lee

**Affiliations:** College of Veterinary Medicine, Jeonbuk National University, Iksan 54596, Republic of Korea; kaithlloren@gmail.com

**Keywords:** oral vaccine, salmonella delivery, porcine circovirus type 2, PCV2d

## Abstract

Oral vaccines are highly envisaged for veterinary applications due to their convenience and ability to induce protective mucosal immunity as the first line of defense. The present investigation harnessed live-attenuated *Salmonella* Typhimurium to orally deliver novel expression vector systems containing the Cap and Rep genes from porcine circovirus type 2 (PCV2), a significant swine pathogen. The antigen expression by the vaccine candidates JOL2885 and JOL2886, comprising eukaryotic pJHL204 and pro-eukaryotic expression pJHL270 plasmids, respectively, was confirmed by Western blot and IFA. We evaluated their immunogenicity and protective efficacy through oral vaccination in a mouse model. This approach elicited both mucosal and systemic immunity against PCV2d. Oral administration of the candidates induced PCV2-specific sIgA, serum IgG antibodies, and neutralizing antibodies, resulting in reduced viral loads in the livers and lungs of PCV2d-challenged mice. T-lymphocyte proliferation and flow-cytometry assays confirmed enhanced cellular immune responses after oral inoculation. The synchronized elicitation of both Th1 and Th2 responses was also confirmed by enhanced expression of TNF-α, IFN-γ, IL-4, MHC-I, and MHC-II. Our findings highlight the effectiveness and safety of the constructs with an engineered-attenuated *S. Typhimurium*, suggesting its potential application as an oral PCV2 vaccine candidate.

## 1. Introduction

Porcine circovirus disease (PCVD), mainly caused by the pathogenic porcine circovirus 2 (PCV2), is one of the most economically important swine diseases associated with several disease syndromes including systemic (PCV-SD), reproductive diseases (PCV-RD), PCV2-subclinical infection (PCV2-SI), and porcine dermatitis and nephropathy syndrome (PDNS) [1]. This small, non-enveloped, single-stranded DNA virus has three types: PCV1, which is considered non-pathogenic, and PCV2 and PCV3, which are pathogenic [2]. Epidemiological analyses revealed genotype shifts in the past decades, where the first genotype shift replaced PCV2a to PCV2b, and the second shift occurred in the past years, with PCV2d currently predominating worldwide [3,4]. The 1700 bp genome of the PCV2 DNA genome consists of two major open reading frames (ORFs), important for its pathogenicity. ORF1 encodes the replicase (Rep) that is related to viral DNA replication, and ORF2 encodes the viral capsid protein (Cap), the sole structural protein, main antigen and major immunogenic polypeptide of PCV2 [5,6,7]. Transmission of PCV2 has been shown primarily by oronasal or via contact with contaminated fomites, feeds, and biological products and through direct contact with infected pigs, including biological excretions such as urine, feces, nasal, ocular, and bronchial, where PCV2 is often shed and detected [1,8,9]. With the high presence of PCV2 in the nasal and fecal excretions of infected animals, this persistent shedding increases the potential transmission from infected to susceptible animals [8,9], affirming the initial entry and spread of PCV2 via the mucosal surface of the gastrointestinal and respiratory tract.

As a multifactorial disease with currently no effective treatment, critical efforts to control PCV2 infections include enhanced biosecurity and vaccination [10]. There are PCV2a-based commercial vaccines available: inactivated PCV2 virus, PCV2 capsid-based subunit vaccines, and inactivated chimeric vaccine [11]. Vaccination with these commercial vaccines has played an important role in the prevention and control of PCV2, especially in reducing viremia, lymphoid tissue damage, and viral replication of PCV2 in pigs [11,12]. However, these licensed vaccines are administered parenterally by intramuscular injection, which may have some limitations in inducing the protective mucosal immunity [13] that is essential for protecting or eliminating infection at the mucosal invasion sites of pathogens such as PCV2 [14,15]. Thus, the development of a mucosal vaccine could further potentiate the approach to vaccination against PCV2 for a broader immune response. Although these PCV2a-based vaccines could confer cross-protection against PCV2b and PCV2d, it is also necessary to develop next-generation vaccines based on the currently predominating genotype PCV2d. Moreover, the industrial applicability of parenteral vaccinations is yet another complication. Oral vaccines that can be formulated as feed or water mixable would be an ideal choice against PCV2 [15]. 

The use of bacteria as vehicles for vaccination has gained interest due to their intrinsic properties, particularly bacterial species that can invade eukaryotic cell types, colonize specific mucosal surfaces, and target inductive cells of the immune system [16,17]. As such, the *Salmonella*-mediated delivery of specific plasmid-encoded heterologous antigens to target eukaryotic host cells and expression of the target immunogen on its surface has been widely demonstrated [18,19]. Evidence has shown these advanced systems to be efficient in successfully transferring eukaryotic expression plasmids or expressing the antigen on its surface for the induction of immune responses against specific pathogens [20,21,22]. Thus, *Salmonella*-mediated vaccination uniquely offers versatility in inducing potent systemic and mucosal responses through mucosal administration such as oral, nasal and ocular, which can especially be ideal for mass vaccination against mucosal and systemic pathogens. The exploitation of these advantages offered by *Salmonella*-based vaccines requires appropriate plasmid vector systems, which are being developed to enhance prokaryotic or eukaryotic antigen expression that consequently improves immune responses. In this study, we utilized our novel dual expression plasmid, p270, which expresses antigens from both eukaryotic and prokaryotic origin as previously demonstrated against omicron SARS-CoV-2, which showed both humoral and cellular immune responses [23].

To reduce the global burden of infectious diseases in both humans and animals, particularly pathogens that infect or transmit via mucosal sites such as PCV2 in pigs, there is a need to develop next-generation vaccines that improve and generate protective immunity at mucosal sites [24]. Thus, the advantages of a *Salmonella*-based oral vaccine, such as the induction of both mucosal and systemic immunity and convenient administration applicable for mass vaccination, offer a potential vaccine strategy against PCV2. Here, we developed a live-attenuated *Salmonella*-based vaccine with novel expression plasmids that express the immunogenic PCV2 Cap and Rep and evaluated its broad-spectrum immune responses and protective efficacy for a potential oral vaccination strategy against PCV2.

## 2. Materials and Methods

### 2.1. Bacterial Strains, Plasmids, Viruses, and Cell Lines

All bacteria and plasmids used in this study are listed in Table 1. A live-attenuated *Salmonella* Typhimurium named JOL2500 with genotype *∆lon ∆cpxr ∆sifA ∆asd* was used as a plasmid carrier as previously described [25] and was grown in LB broth or LB agar plates (BD, Franklin Lakes, NJ, USA) at 37 °C. For cloning and challenge experiments in mice, a PCV2d clinical isolate from South Korea PCV2d/CBNU0324 (GeneBank accession number MN545963) was used and propagated in porcine kidney cells (PK-15) maintained in 5% FBS-supplemented DMEM (Lonza, Walkersville, MD, USA) with penicillin and streptomycin. Murine macrophage cells (RAW 264.7) were used for in vitro studies and were maintained in 10% FBS-supplemented DMEM at 37 °C with 5% CO_2_.

### 2.2. Design and Development of the Vaccine Candidates

The *cap* and *rep* genes were selected as the target antigens for the eukaryotic expression and were amplified from the PCV2d isolate using the primers listed in Table 1. The *cap* and *rep* genes were linked by a self-cleaving peptide with 66 base pairs encoding 22 amino acids of porcine teschovirus-1 called P2A for a multi-antigen delivery [28]. For the prokaryotic expression, the PCV2 capsid sequence was codon optimized for *Salmonella* Typhimurium (ST) and was custom synthesized (Cosmogenetech, Seoul, Republic of Korea). The amplified inserts, Cap-P2A-Rep for eukaryotic expression and only Cap for prokaryotic expression were inserted into the suitable MCS region of the novel dual-expression plasmid, p270, previously designed with cytomegalovirus (CMV) promoter for eukaryotic and Ptrc promoter for prokaryotic antigen expression [23]. This multi-antigen, dual-expressing vaccine construct is designated as p270:EuCap-Rep+ProCap. Additionally, a eukaryotic expression plasmid, p204, a Semliki-based vector as previously designed [25], serves as a baseline for comparison and was inserted with the Cap-P2A-Rep construct only and is designated as p204:Cap-Rep. All constructs were verified by sequencing before electroporation into a live-attenuated *Salmonella* Typhimurium JOL2500 (*∆lon ∆cpxr ∆sifA ∆asd*) for vaccine delivery resulting in JOL2885 (p204:Cap-Rep) and JOL2886 (p270:EuCap-Rep+ProCap).

### 2.3. Confirming the Expression by RT-PCR, Immunofluorescence and Western Blot

To confirm the eukaryotic expression by the vaccine constructs, JOL2885 and JOL2886 were used for bactofection in RAW cells for 3 h. After killing extracellular bacteria with 100 μg/mL gentamycin, cells were further incubated for 48 h at 37 °C with 5% CO_2_. For confirmation by RT-PCR, infected cells were harvested for total RNA extraction using Trizol method, and the synthesized cDNA was then mixed with the gene-specific primers listed in Table 1 for PCR and agarose gel analysis. Furthermore, eukaryotic expression was visualized by immunofluorescence using the primary antibodies raised in rabbits at 1:200 dilution as previously described [29]. After washing the cells, AlexaFluor 488 secondary antibody (Invitrogen, Waltham, MA, USA) was added and incubated for 1 h. The immunostained cells were then viewed under the fluorescence microscope to detect positive green fluorescence of the target antigens Cap and Rep. Additionally, to detect the expression of the antigens at the protein level, cell lysates from RAW cells were mixed with SDS sample buffer and run on 12% gel. After trans-blot, the PVDF membrane was blocked with 5% skim milk for 2 h and then incubated with primary antibodies raised in rabbits at 1:200 dilution overnight at 4 °C. The membranes were then thoroughly washed with PBST before incubating with ant-rabbit IgG-HRP at 1:6000 and further incubated for 1 h. For the prokaryotic expression confirmation by JOL2886 (p270:EuCap-Rep+ProCap), the strain was cultured to OD_600_ of 1, and the bacterial lysate was collected. The proteins were separated in 12% SDS-PAGE gel and analyzed as similarly described above.

### 2.4. Mice Immunization, PCV2d Virus Challenge and Sampling

5-week-old SPF female BALB/c mice (Samtako, Osan-si, Republic of Korea) were housed in a laboratory animal facility and provided with fresh water and feed. All animal experiments were performed according to the guidelines for laboratory animal care and use and were approved by Jeonbuk National University Animal Ethics Committee (NON2022-024-001). To evaluate the immunoprotectivity of the vaccine candidates used as an oral vaccine, randomly grouped mice (*n* = 6) were orally immunized four times with 100 μL 10^8^ CFU of JOL2885, JOL2886 or PBS given at 1-week intervals. In comparison, the candidates were also inoculated into groups of mice by intramuscular route given twice with 100 μL 10^7^ CFU at 2-week intervals. After primary immunization, serum samples were collected on days 14 and 21 for specific antibody detection by ELISA and neutralizing antibodies. Three mice from each group were sacrificed 3 weeks after the last booster to collect the splenocytes for assessment of T cell populations by flow cytometry. Two weeks after the last booster, all immunized mice were challenged intraperitoneally with PCV2d isolate (0.2 mL of 10^4.5^TCID50/mL) propagated in PK-15 cells [30,31]. Three weeks later, liver and lungs were collected from three test animals in each group for PCV2 titer determination by qPCR and immunohistochemical detection of PCV2.

### 2.5. Enzyme-Linked Immunosorbent Assay (ELISA)

To evaluate the induction of IgG and sIgA, serum, lungs, and intestinal wash samples were collected from immunized mice. Indirect ELISA was performed as described previously [32] with slight modifications. Briefly, 96-well ELISA plates were coated with 100 μL of Cap or Rep purified protein diluted to a final concentration of 2 μg/well in coating buffer (9.6 pH) and incubated overnight at 4C. Serum samples were 2-fold serially diluted, added to test wells, and incubated for 1 h at 37 °C. After washing thrice with PBST (0.5% Tween-20), IgG, IgG1, IgG2a, and sIgA titers against the specific PCV2 antigens were measured by incubating the plates with HRP-conjugated goat anti-mouse IgG, IgG1, IgG2a, or IgA antibody (Southern Biotech, Birmingham, AL, USA) at 1:3000 dilution for 1 h at 37 °C. After final washing with PBST, OPD substrate (Sigma-Aldrich, St. Louis, MO, USA) was used to develop the signal, and the OD values were read at 492 nm using an ELISA plate reader (Tecan, Männedorf, Switzerland).

### 2.6. Virus Neutralization Assay

Neutralizing antibodies were measured at week 2 after the last booster by virus-neutralizing activity assay. Briefly, heat-inactivated serum samples at 56 °C for 30 min were 2-fold serially diluted and incubated with equal volumes of 200 TCID_50_ PCV2d isolate for 1 h at 37 °C. The serum–virus mixture was added to 50% confluent PK-15 cells plated in 96-well plates and further incubated for 72 h at 37 °C with 5% CO_2_. The culture plate was then fixed with 80% acetone at −20 °C for 15 min and blocked with 5% BSA for 1 h at 37 °C. Cells were then washed with PBS and incubated with anti-Rep polyclonal antibody followed by Alexa Fluor 488-conjugated donkey anti-rabbit IgG (Invitrogen, Waltham, MA, USA) as the secondary antibody. The cells were viewed under a fluorescence microscope, and serum titers were expressed as the highest serum dilution resulting in ≥70% reduction in infected cell cultures.

### 2.7. Lymphocyte Proliferation Assay

Splenocytes collected from immunized mice were subjected to MTT assay as previously described [33] with slight modification to determine the cell proliferation. Briefly, splenocytes were cultured in RPMI 1640 (Lonza, Walkersville, MD, USA) containing 10% FBS and 1% penicillin-streptomycin and were seeded into 96-well plates at 1 × 10^5^ cells/well. Purified PCV2 Cap and Rep proteins prepared at a final concentration of 2 μg per well with 1 μg each protein was added to stimulate splenocytes. After 48 h of incubation at 37 °C in 5% CO_2_, 10 μL of MTT (5 mg/mL) (Sigma-Aldrich, St. Louis, MO, USA) was added to each well and further incubated for 4 h before solubilizing with DMSO (Sigma-Aldrich, St. Louis, MO, USA). The absorbance was measured at 570 nm, and the stimulation index was calculated by dividing the values from stimulated cells with that of unstimulated cells.

### 2.8. Flow Cytometric Analysis and Cytokine Expression

CD4^+^ and CD8^+^ differentiation of T cells from stimulated splenocytes as similarly described above in lymphocyte proliferation were incubated with CD3e-PE, CD8a-FITC, and CD4-PerCPVio700 antibodies (Miltenyi-Biotec, Bergisch Gladbach, Germany) at 4 °C for 30 min in dark. Additionally, to compare the induction of MHCI and MHCII molecules, FITC labeled MHC class 1 (H-2Kb) and MHC class II (I-A/I-E) FACS analysis markers (Bio-Rad Laboratories, Inc., Hercules, CA, USA) were used according to the manufacturer’s protocol. Cells were washed and resuspended with MACSQuant running buffer (Miltenyi-Biotec, Bergisch Gladbach, Germany) before flow cytometric analysis. The percentage of CD3^+^CD4^+^, CD3^+^CD8^+^ T cells, MHCI, and MHCII was analyzed using a benchtop flow cytometer, MACSQuant^®^ analyzer (Miltenyi-Biotec, Bergisch Gladbach, Germany).

Moreover, stimulated splenocytes in 24-well plates were harvested and total RNA was isolated. cDNA was then synthesized using reverse transcription master premix (Elpis Biotech, Daejeon, Republic of Korea) with oligo d(T)15 primer according to the manufacturer’s protocol. Levels of IFN-γ, TNF-α, and IL-4 mRNA were analyzed by qPCR using the primers listed in Table 1. The relative cytokine expression levels were determined by the 2^−∆∆CT^ method using β-actin as the housekeeping gene.

### 2.9. Detection of PCV2 in Mice Tissues by qPCR and Immunohistochemistry

PCV2 DNA was quantified from the liver and lungs of mice at day 21 post-challenge by real-time quantitative PCR as previously described [26] using the primers listed in Table 1. Real-time PCR was carried out using 2x SYBR Green qPCR Master Mix (Elpis Biotech, Daejeon, Republic of Korea) according to the manufacturer’s instructions and performed in Applied Biosystems^®^ StepOnePlus Real-Time PCR system. The viral copies from each sample were calculated based on the standard curve generated from serial dilutions of a plasmid containing the Cap gene. 

Furthermore, immunohistochemistry (IHC) was performed as previously described [34] with slight modifications. Briefly, mice organs such as the liver, lungs, and mesenteric lymph node were fixed in 10% neutral-buffered formalin, dehydrated, embedded, and sectioned at 4 µm. Slides were then deparaffinized and dehydrated in a series of diluted ethanol before incubating the slides in antigen retrieval buffer for 20 min. Inhibition of endogenous peroxidase activity was done by adding 30% H_2_O_2_ in methanol for 30 min. After the slides were blocked with 5% BSA at room temperature for 1 h, the slides were incubated with PCV2 Cap polyclonal antibody (1:50) at 4 °C overnight. After washing with PBST, an anti-rabbit biotinylated secondary antibody (1:1000) (Southern Biotech, Birmingham, AL, USA) was added and incubated at room temperature for 1 h. The slides were then incubated with diaminobenzidine (DAB) hydrogen peroxide solution for 3 min and then stained with methylene green for 3 min. The slides were mounted and viewed under the microscope.

### 2.10. Statistical Analysis

GraphPad Prism 10.0.0 was used in the statistical analysis. Two-way ANOVA was used to determine the differences between the groups. The data are presented as mean ± SEM. A *p*-value of <0.05 was considered significant.

## 3. Results

### 3.1. Vaccine Construction and Confirmation of Eukaryotic and Prokaryotic Expression of Target Antigens

**Figure 1:**
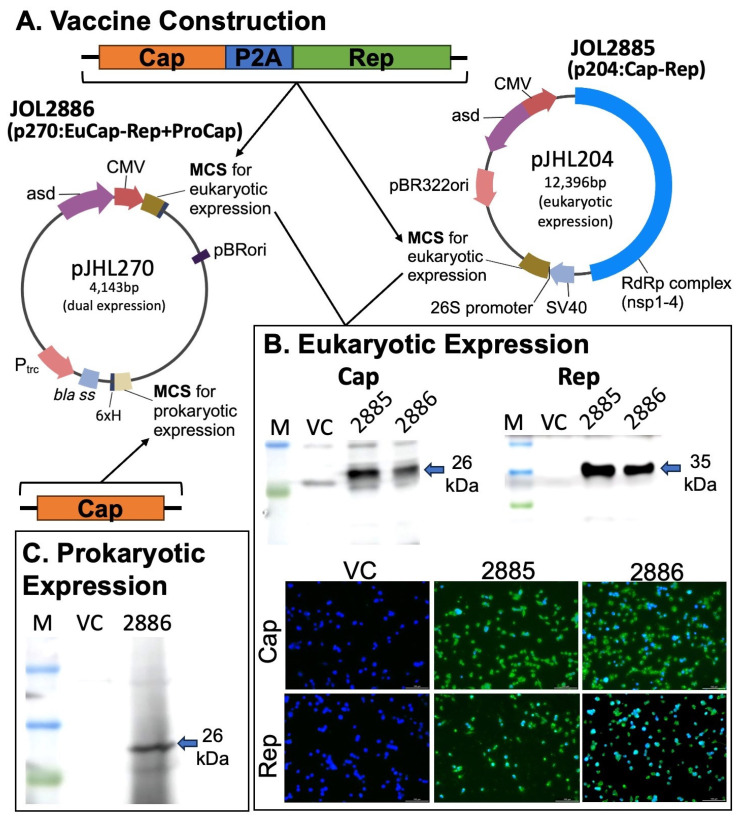
Schematic representation of the vaccine design, construction, and expression confirmation of the target antigens. (**A**) Schematic diagram of the eukaryotic expression plasmid (pJHL204) and dual-expression plasmid (pJHL270) illustrating the plasmid map, key components, and strategy for vaccine construction. The Cap and Rep cloned from PCV2d were linked by a self-cleaving peptide, P2A, and were inserted into the MCS region under the CMV promoter for eukaryotic expression in pJHL204 and pJHL270. An optimized sequence of Cap was inserted into the MCS region under the Ptrc promoter for prokaryotic expression in pJHL270. (**B**) Eukaryotic expression validation of Cap and Rep by the vaccine strains JOL2885 (p204:Cap-Rep) and JOL2886 (p270:EuCap-Rep+ProCap) through immunofluorescence and Western blot detection of Cap and Rep in transfected RAW 264.7 cells using hyperimmune rabbit antisera raised against the target proteins. Green fluorescence indicates positive expression of the respective proteins, and cell nuclei were stained with DAPI as shown by blue fluorophore. (**C**) Western blot validation of Cap expression by the prokaryotic side of JOL2886 (p270:EuCap-Rep+ProCap).

**Figure 2:**
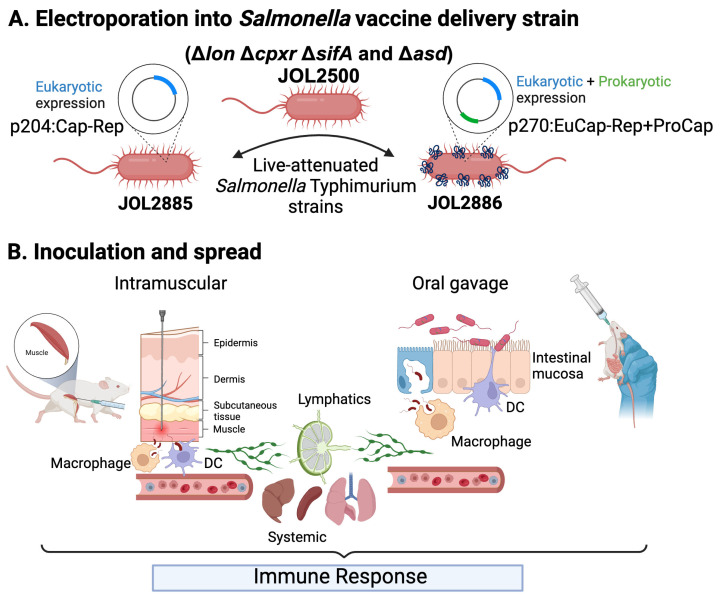
Schematic diagram of *Salmonella*-based vaccine delivery. (**A**) Graphical representation of live-attenuated *Salmonella* with genotype *∆lon*, *∆cpxr*, *∆sifA*, *∆asd* electroporated with the constructed plasmids for bacterial-based vaccine delivery. (**B**) Vaccine delivery via intramuscular or oral administration, invasion of macrophages and dendritic cells (Antigen-presenting cells), and further spread through the bloodstream and lymphatics into different organs for induction of immune responses. The figure was created using BioRender online tool (https://app.biorender.com/ (accessed on 14 October 2023)).

Two vaccine candidates were designed and constructed using two expression plasmids: p204 for eukaryotic expression only and p270, a dual-expression vector for both eukaryotic and prokaryotic expression (Figure 1A). The target antigens for eukaryotic expression were Cap and Rep of PCV2d, which were amplified and connected by a self-cleaving peptide P2A and were inserted between the respective restriction sites in the multiple cloning site (MCS) region of the p204 or the p270 under the control of the cytomegalovirus (CMV) promoter. In addition, a Cap gene was inserted into the MCS of p270 under the control of the Ptrc promoter for prokaryotic expression (Figure 1A). The resulting plasmids, p204:Cap-Rep and p270:EuCap-Rep+ProCap, were then constructed and electroporated into a live-attenuated *Salmonella* Typhimurium JOL2500 (*∆lon ∆cpxr ∆sifA ∆asd*), generating JOL2885 and JOL2886, respectively (Figure 2A). The eukaryotic expression of these two proteins was validated by Western blot analysis, which revealed bands at the expected size for Cap at 26 kDa and Rep at 35 kDa, showing the efficient expression of the target proteins by the candidates (Figure 1B). Subsequently, the eukaryotic expression was also confirmed in vitro using murine macrophage cells by immunofluorescence assay and revealed positive green fluorescence of Cap and Rep proteins in the vaccine-transfected cells, demonstrating efficient antigen delivery by the live-attenuated *Salmonella* and by the expression plasmids (Figure 1B). Moreover, the prokaryotic expression of Cap constructed into the dual expression plasmid, p270, was also confirmed by Western blot analysis and showed the expected band at 26 kDa (Figure 1C).

### 3.2. Humoral and Mucosal Immune Responses Induced by the Salmonella-Delivered Vaccines

**Figure 3:**
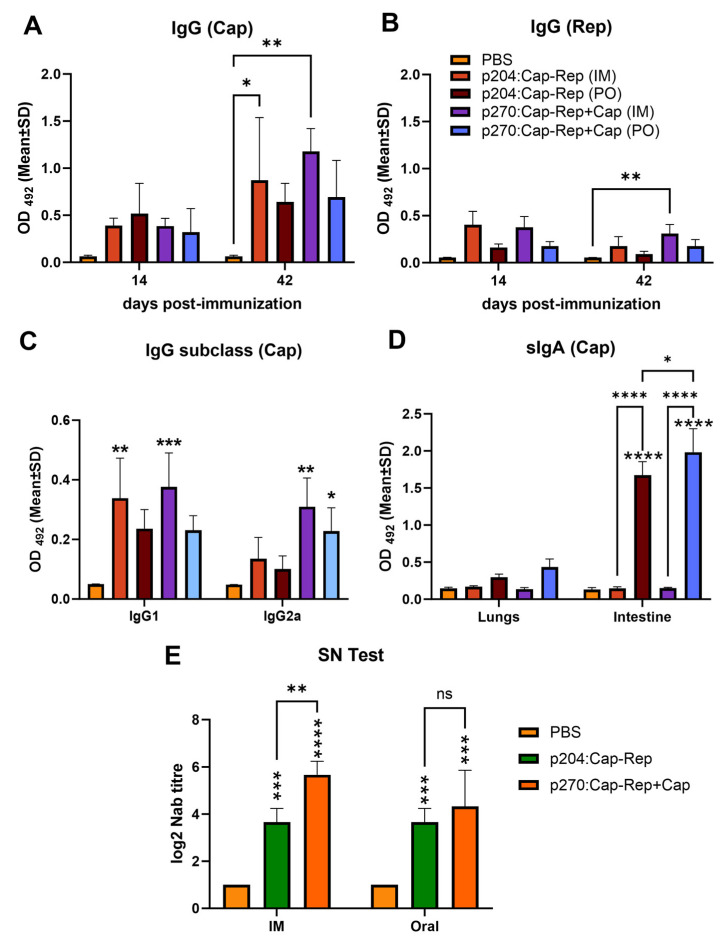
Induction of humoral and mucosal immune responses by JOL2885 (p204:Cap-Rep) and JOL2886 (p270:EuCap-Rep+ProCap) in BALB/c mice. Groups of mice were given two doses of 1 × 10^7^ CFU intramuscularly at 2-week intervals, whereas other groups of mice were given four doses of 1 × 10^8^ CFU orally at 1-week intervals. (**A**,**B**) IgG level was assessed in the serum of immunized mice at 14 and 42 days after primary immunization by ELISA with Cap or Rep purified proteins as capture antigens. (**C**) IgG1 and IgG2a subclass were assessed in the serum of immunized mice at 42 days post-immunization using Cap purified protein as capture antigen. (**D**) The secretory IgA (sIgA) level in the lung homogenate and intestinal wash was assessed by ELISA using Cap purified protein as capture antigen. (**E**) Neutralizing antibody titer (NAb) at day 42 after primary immunization was quantified by serum neutralization (SN) test and analyzed by end-point dilution reduction assay. The SEM is denoted by the error bars. The data were analyzed by two-way ANOVA. ^ns^ *p* > 0.05, * *p* < 0.05, ** *p* < 0.01, *** *p* < 0.001 and **** *p* < 0.0001.

To evaluate whether the developed vaccines could induce humoral and mucosal antibody responses, anti-PCV2-specific IgG, IgG1, IgG2a, and secretory IgA (sIgA) antibodies were detected by ELISA using Cap or Rep purified proteins as coating antigens at days 14 and 42 post-immunization. The results showed that all the *Salmonella*-vaccinated groups by IM or PO (Figure 2B) induced high levels of IgG Cap- and Rep-specific antibodies, especially at day 42 post-immunization, compared to the PBS group (negative control), where specific antibodies were undetectable (Figure 3A,B). Particularly, JOL2886 (p270:EuCap-Rep+ProCap) given IM induced significantly high levels of IgG Cap-specific antibodies followed by IM inoculation of JOL2885 (p204:Cap-Rep) compared to the PBS group. The oral inoculation, however, also induced high levels of IgG compared to the PBS group and no significant difference compared to the IM inoculation, indicating that oral inoculation of the candidates is also comparable to that of IM. IgG1 and IgG2a were also assessed. IgG1 was significantly high in both vaccine candidates when given IM, and oral administration also elicited a considerable increase in IgG1 compared to the PBS group (Figure 3C). IgG2a was significantly increased in mice vaccinated either IM or orally with JOL2886 (p270:EuCap-Rep+ProCap) compared to the PBS group (Figure 3C). Overall, there is a balance in the elicited IgG1/IgG2a, particularly with JOL2886 (p270:EuCap-Rep+ProCap). Conversely, the mucosal immune response was significantly induced by the oral inoculation of JOL2885 (p204:Cap-Rep) and JOL2886 (p270:EuCap-Rep+ProCap) compared to IM inoculation, as shown by the sIgA levels in the intestine (Figure 3D). Additionally, comparing the two candidates for mucosal immune response, PO inoculation of JOL2886 induced significantly higher sIgA than JOL2885 (Figure 3D), suggesting the probable added contribution of the prokaryotic expression of Cap by JOL2886 in inducing immune responses. 

To assess whether the antibodies elicited by the immunized mice could neutralize the virus, a serum neutralization (SN) assay was performed using the serum collected at day 41 post-immunization against PCV2d virus isolate. The results indicated that all IM- or PO-immunized groups generated neutralizing antibodies, except the PBS group (Figure 3E). Specifically, both IM and PO inoculation of JOL2885 (p204:Cap-Rep) induced a mean of 1:3.6 neutralizing antibody (NA) titer, whereas IM or PO inoculation of JOL2886 (p270:EuCap-Rep+ProCap) induced a mean of 1:5.66 NA titer and 1:4.33 NA titer, respectively. There was no significant difference between the neutralizing antibodies generated by IM or PO inoculation, indicating that oral inoculation similarly elicited neutralizing antibodies with that of the IM.

### 3.3. CD4^+^ and CD8^+^ T Cell Differentiation and Lymphocyte Proliferative Response

**Figure 4:**
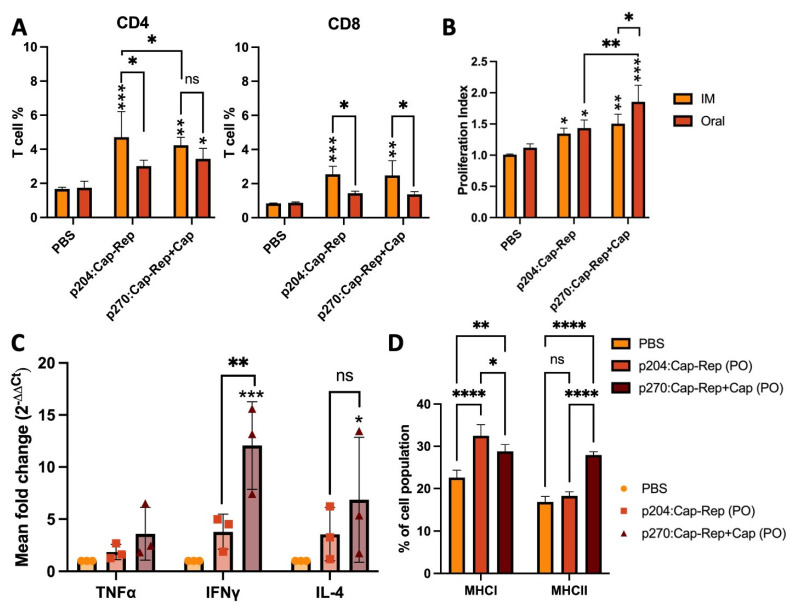
Cell-mediated immune responses by JOL2885 (p204:Cap-Rep) and JOL2886 (p270:EuCap-Rep+ProCap) in immunized BALB/c mice. Three weeks post-immunization, collected splenocytes were stimulated with purified Cap and Rep proteins for 48 h. (**A**) Percentages of CD4^+^ and CD8^+^ T cells presented in a bar diagram as analyzed by flow cytometry. (**B**) Bar diagram showing splenocyte proliferation index after stimulation with Cap and Rep proteins. (**C**) Changes in the cytokine expression profile of IFN-γ, TNF-α, and IL-4 in orally immunized mice were determined by qPCR. (**D**) Bar diagram showing the percentage of MHCI and MHCII molecules. The data were analyzed by two-way ANOVA. ^ns^ *p* > 0.05, * *p* < 0.05, ** *p* < 0.01, *** *p* < 0.001 and **** *p* < 0.0001.

To further characterize the immune responses induced by the vaccine candidates, the cell-mediated immune response was assessed by flow cytometry and proliferation assay of the collected splenocytes at week 3 post-immunization that were stimulated with Cap and Rep recombinant proteins. Both CD4^+^ and CD8^+^ T cell differentiation was relatively increased in all immunized mice compared to the PBS group with significantly higher CD4^+^ and CD8^+^, particularly in IM-inoculated mice with JOL2885 or JOL2886 than oral inoculation (Figure 4A). Interestingly, JOL2886 (p270:EuCap-Rep+ProCap), when inoculated orally, has also significantly higher induced CD4^+^ T cells than the PBS group, and there was no significant difference in the CD4^+^ T cell differentiation from the PO-inoculated JOL2886, suggesting that oral immunization with JOL2886 could also induce a high cell-mediated immune response. 

Moreover, lymphocyte proliferative responses were significantly higher in all immunized groups compared to the PBS group (Figure 4B) after stimulation with the Cap and Rep recombinant proteins. Stimulated lymphocytes from orally immunized mice with JOL2886 showed the highest significant proliferation, followed by IM-immunized mice. Comparing the proliferation index between the two candidates, JOL2886 showed a higher index than JOL2885, whereas oral immunization showed a higher index than IM (Figure 4B).

### 3.4. Cytokine Expression Profile and MHC Class I and MHC Class II Molecules Stimulation

The cytokine expression in the stimulated lymphocytes was also evaluated by quantitative real-time PCR to determine the expression patterns of the cytokines TNF-α, IFN-γ, and IL-4 in PO-immunized mice. Oral immunization with JOL2886 (p270:EuCap-Rep+ProCap) showed significantly more IFN-γ than JOL2885 (p204:Cap-Rep) and PBS group (Figure 4C). JOL2886 also had significantly more IL-4 than the PBS group. The results demonstrate the induction of the Th1 (TNF-α, IFN-γ) and Th2 (IL-4) immune responses, which are important for the adaptive immune response and for regulating antibody production, by the candidate vaccines, particularly by JOL2886 (p270:EuCap-Rep+ProCap). 

Furthermore, MHC class I and class II regulation by immunization with the candidate vaccines were evaluated by flow cytometry. MHC-I+ cells percentage in stimulated splenocytes of mice immunized orally was significantly higher in JOL2885 (p204:Cap-Rep), followed by JOL2886 (p270:EuCap-Rep+ProCap), compared to the PBS group (Figure 4D). JOL2885 showed a significantly higher MHC-I+ cell percentage than JOL2886. Conversely, for MHC-II+ cell stimulation, JOL2886 had a significantly higher percentage than JOL2885 and the PBS group. The results showed that JOL2885, carrying a eukaryotic plasmid, tends to incline towards MHC-I+ cell stimulation, whereas JOL2886, carrying a dual-expression plasmid for eukaryotic and prokaryotic expression, could stimulate both MHCI-I+ and MHC-II+ cells (Figure 4D).

### 3.5. Detection of PCV2 in Virus-Challenged Mice

**Figure 5:**
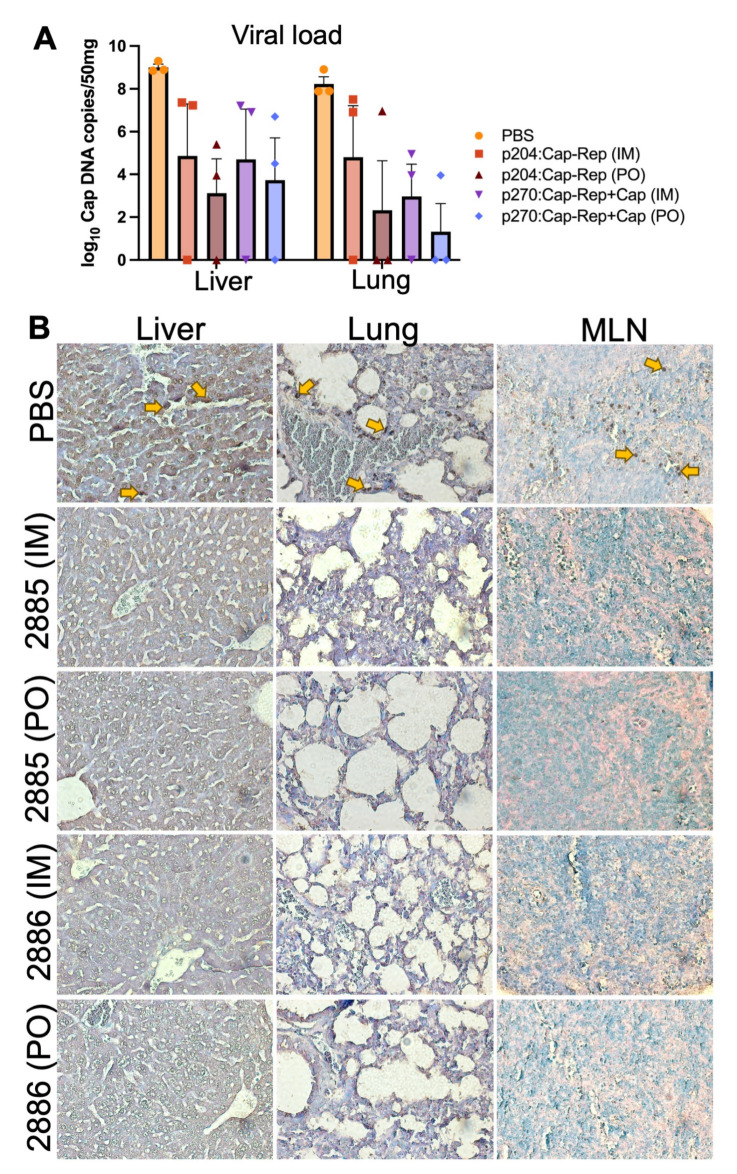
Viral load in the organs of vaccinated mice after PCV2d challenge. Groups of mice were immunized with 2 doses of 1 × 10^7^ CFU intramuscularly at 2-week intervals or with 4 doses of 1 × 10^8^ CFU orally at 1-week intervals and then challenged with PCV2d intraperitoneally. (**A**) PCV2 Cap genomic copies in the liver and lungs of vaccinated mice 21 days after PCV2d challenge. (**B**) Representative images of immunohistochemical detection of PCV2 in liver, lungs, and spleen of PCV2d-challenged mice. Positive immunolabeling for PCV2 antigen was indicated by a dark brown signal (yellow arrow). All images were taken at 400× magnification. The bars represent the mean values and the SEM is denoted by the error bars. The data were analyzed by two-way ANOVA.

Different tissues collected from all post-challenged experimental groups were used for PCV2 DNA extraction and immunohistochemical detection of PCV2 to evaluate the protective efficacy of the developed vaccines in the murine model. As shown in Figure 5, the PBS group showed the highest viral load compared to all other groups. The amounts of virus in the liver and lungs of the immunized groups either by IM or PO were lower compared to the PBS group, and there was no detection of PCV2 in 1 or 2 out of 3 challenged mice in each group (Figure 5). Particularly, mice orally immunized with JOL2886 (p270:EuCap-Rep+ProCap) showed the lowest viral load in the lungs (Figure 5A). Additionally, PCV2 was also detected in the organ tissues by immunohistochemistry. Liver, lungs, and MLN tissue sections from the PBS group showed significantly higher amounts of PCV2 antigens, as shown by the intense brown signals, compared to the vaccinated groups where no-to-less signals were observed (Figure 5B).

## 4. Discussion

PCV2 causes several disease syndromes in pigs resulting in significant economic losses in the swine industry globally. In this study, the oral vaccination of the vaccine candidates elicited a broad spectrum of immunity, covering both systemic and mucosal sites through the safe and efficient vaccine delivery of the *Salmonella* vaccine strain to different organs such as liver, spleen, and lungs [29,35,36] and expression of the target antigens, Cap and Rep, in antigen-presenting cells (Figure 2). *Salmonella* Typhimurium is one of several bacterial species that can transfer eukaryotic expression plasmids into host cells, which can serve as a carrier for vaccine antigens or its genetic material [37,38]. Several studies have also demonstrated that oral vaccination with a live-attenuated *Salmonella* Typhimurium carrying immunogenic and protective homologous antigens of the target pathogens can induce humoral, cellular, and mucosal immune responses as well as protect animal models from challenges with either human or animal pathogens [18,39,40,41]. Parenteral vaccines demand a complex production process, necessitate an expensive cold chain for safeguarding vaccine effectiveness, mandate substantial quantities of sterile resources, and rely on a considerable workforce for administering them to numerous animals, leading to elevated expenses [42]. In contrast, oral vaccines offer convenience, cost-effectiveness, and broad industrial applicability [43]. They trigger the host’s mucosal immune system, the body’s largest reservoir of immune cells, to generate substantial quantities of targeted protective antibodies against the virus [44,45,46]. With the ability of *Salmonella* to invade the mucosal-associated lymphoid tissues in the gut through oral inoculation (Figure 2B), it improves the stimulation of mucosal immunity and induces sIgA [17,47], which may be critical in protecting against some pathogens such as PCV2. Thus, the use of live-attenuated *Salmonella* as a carrier for DNA vaccines for oral vaccination has gained interest due to its multiple advantages.

Although PCV2 infection in mice does not similarly resemble that of pigs, several studies have shown that PCV2 can replicate in some mouse strains, including BALB/c mouse [48], which is one of the extensively used animal models for the PCV2 vaccine studies [32,34,49,50,51]. Secretory IgA (sIgA) in the BALB/c mouse model has been well recognized as an important first line of defense for protection against pathogens in the mucosal surfaces and thus, plays an important role in mucosal immunity [52]. Studies have shown that the production of sIgA depends on the uptake and processing of immunogens by the antigen-presenting cells in Peyer’s patch M cells and the activation of T-cells and B-cell class switch [53]. In connection to that, our results demonstrated that even though the serum IgG antibodies were highly elicited by IM inoculation of the vaccine candidates, the oral administration of the vaccine candidates significantly induced high PCV2-specific sIgA antibody levels, particularly in the intestine. This induction of mucosal immunity could imply an important aspect, especially when considering the nature of infection of the target pathogen, PCV2, which is generally transmitted through the oronasal route [9]. Also, even though the IM inoculation showed induction of higher levels of IgG antibodies than PO, there is no significant difference in the antibody levels induced by the IM or PO inoculation, which suggests that oral inoculation also induces comparable antibodies. Additionally, the analysis of IgG1 and IgG2a in the serum of immunized mice showed that the JOL2885 (p204:Cap-Rep) induced a Th2-biased immune response, whereas JOL2886 (p270:EuCap-Rep+ProCap) induced balanced Th1 and Th2 immune responses (Figure 3C). Th1 cells secrete IFN-γ and TNF-α, which promote macrophage activation and cytotoxic T lymphocyte proliferation, and stimulate immunoglobulin class switching in B cells for the production of IgG2a antibodies, leading to the clearance of viruses and other pathogens [51,54]. On the other hand, Th2 cells predominantly produce IL-4 and IL-10, which play an important role in the activation of the humoral immune response by B cell proliferation and induce isotype switching to IgG1 [55,56]. Thus, the induction of Th1/Th2 immune responses is essential in the protective efficacy of the candidate vaccines. 

In addition, the presence of PCV2-neutralizing antibodies has also been associated with enhanced protection against PCV2 infection [57]. This was similarly observed in the present study, wherein significantly high neutralizing antibodies were elicited by the oral administration of the candidate vaccines, which correlated with the outcomes of relatively decreased viral load and the presence of PCV2 in the liver and lungs of the PCV2-challenged mice (Figure 5). Also, no significant difference in the neutralizing antibodies induced by IM or PO inoculation was observed, suggesting that the oral inoculation similarly elicited high neutralizing antibodies as IM inoculation. 

Moreover, the participation of T-cell activation by vaccination is valuable in cellular immunity. The CD4^+^ T cells are helper cells that respond to exogenous antigens as presented by MHC class II molecules, whereas CD8+ T cells are cytotoxic T cells that respond to endogenous antigens presented by MHC class I molecules [58,59]. The regulations brought by the activation of these MHC pathways and subsequent changes in the T cell population are often used as indicators of immune status. Our results revealed that the oral administration of the candidate vaccines could also increase the T-cell population, although not as high when administrated intramuscularly. The impairment of immune cells and cytokine balance have been responsible for PCV2 infections in pigs [60]. In this study, splenocytes from vaccinated mice were shown to respond well to recall PCV2 Cap and Rep antigens by releasing high levels of IFN-γ and IL-4 cytokines (Figure 4C), which are typically downregulated in PCV2-infected pigs [61]. Therefore, the induction of IFN-γ and IL-4 by the candidate vaccines appears to be an important correlation to immunity and protection against PCV2 [61]. 

The exploitation of a *Salmonella*-mediated vaccine delivery system offers versatility as it can be coupled with advanced vector systems such as plasmid-encoded heterologous antigens for eukaryotic or prokaryotic expression [16]. In the present study, we utilized a eukaryotic expression plasmid, p204, and a dual-expression plasmid, p270, for designing and constructing two vaccines containing the Cap and Rep of PCV2d as the target immunogens (Figure 1). The PCV2 Cap protein has been known to be the major immunogenic structural protein of the virus [6,62] and, thus, is selected to be in both the eukaryotic and prokaryotic expression sides of the dual-expression plasmid, whereas the Rep protein, involved in virus replication [63], was only included for eukaryotic expression. The dual-expression plasmid, p270, coupled with a live-attenuated *Salmonella* JOL2500, allows the prokaryotic expression of the Cap protein through the Ptrc promoter, as well as eukaryotic expression of Cap and Rep through the CMV promoter for efficient vaccine delivery, as confirmed by the Western blot and IFA analysis (Figure 1). Thus, utilizing a dual-expression plasmid in a *Salmonella*-based vaccine delivery advances the approach to antigen delivery, as it could present both exogenous and endogenous antigens for efficient presentation of immunogens into the host immune machinery [37]. Compared with the eukaryotic expression of the antigens by p204, which demonstrated increased activation of the MHC class I, following the processing of the endogenous antigens (plasmids containing the target genes), the p270 was able to activate not just MHC class I but also MHC class II through the addition of an exogenous antigen expressed by the prokaryotic side of its expression system, as observed in our results (Figure 4D), which could have contributed to the higher humoral and mucosal immune responses than p204, and thus, may be beneficial for the induction of broad immunity by oral vaccination. The addition of Cap into the prokaryotic expression side of the candidate vaccine JOL2886 showed generally higher IgG, sIgA, neutralizing antibodies, and cell-mediated immune responses than JOL2885, suggesting that bacterial expression of PCV2 Cap antigen could improve the induction of immune responses in the mucosal and systemic systems.

In conclusion, our results revealed that oral vaccination with an engineered live-attenuated *S. Typhimurium* for delivery of a dual-expression plasmid carrying PCV2 antigens Cap and Rep effectively induced broad-spectrum immunity including systemic and mucosal immunity against PCV2. This protective mucosal immunity brought by oral vaccine inoculation may also be deemed pivotal in achieving a broader immune response, which includes immune responses in the mucosal sites for the local elimination of the virus, in addition to humoral and cellular immunity. Oral vaccination of a bacterial-based vaccine offers many advantages such as low cost, ease of administration, and safety. With such advantages, along with its unique vaccine delivery system, this type of vaccine has potential applications for mass vaccination, especially in the large-scale pig industry. However, concerns about the interference by pre-existing antibodies to *Salmonella* pose some limitations on the application of the *Salmonella* vaccine vector in the field [64]. Several studies have been conducted to examine the effect of pre-existing immunity in the host against the *Salmonella* vector [64]. Some studies concluded that the *Salmonella* vector leads to stronger immune responses [65,66,67], whereas others observed reduced immune responses against the delivered heterologous antigen due to pre-existing immunity against *Salmonella* [68,69]. To diminish the impact of pre-existing immunity on the vaccine delivery efficiency and immunogenicity, an attenuated *Salmonella* vector strain was previously engineered by deleting the O-antigen ligase (*rfal*) from the *Salmonella* Typhimurium genome [70]. The optimized *Salmonella* vaccine strain JOL3000 with genotype *Δlon, Δcpxr, Δrfal, ΔpagL::lpxE, Δasd* [36,71] can be utilized for further studies to explore its efficient vaccine delivery by overcoming the pre-existing antibodies to *Salmonella* and potential application in the swine model. Aside from this, optimizing the dosage, vaccination schedule, and incorporation of stimulators for the host immune response could also be explored to further improve its immunogenicity. Nevertheless, the development of an oral type of vaccine exploiting the advantages of a live-attenuated *Salmonella* Typhimurium as a carrier advances its potential use against various human and animal diseases, such as PCV2.

## Figures and Tables

**Table 1:** List of plasmids, bacterial strains, and primers used in the study.

Plasmid/Bacteria/Primer	Description	Reference
Plasmids		
pET28a(+)	IPTG-inducible expression vector; Kanamycin resistance	Novagen, USA
pJHL204	asd+, CMV promoter, RdRp complex, SV40 promoter, pBR322 ori	[25]
pJHL270	asd+, CMV eukaryotic promoter, Ptrc prokaryotic promoter, pBR322 ori	[23]
pJHL204:Cap-Rep	asd+, CMV promoter, RdRp complex, SV40 promoter, pBR322 ori, Cap-P2A-Rep	This study
pJHL270:EuCap-Rep+ProCap	asd+, CMV eukaryotic promoter, Cap-P2A-Rep, Ptrc prokaryotic promoter, Cap, pBR322 ori	This study
*S. Typhimurium*		
JOL2500	*Salmonella* Typhimurium with genotype ∆lon ∆cpxr ∆sifA ∆asd	Lab stock
JOL2885	JOL2500 carrying pJHL204:Cap-Rep	This study
JOL2886	JOL2500 carrying pJHL270:EuCap-Rep+ProCap	This study
*E. coli*		
DH5α	*E. coli* F^-^Φ80dlacZ∆M15∆ (lacZYA-argF) U169recA1 endA1 hsdR17(rk-, mk^+^) phoA supE44 thi1 gyr A96 relA1λ-	Lab stock
JOL2873	DH5α carrying pET28(a) + Cap	Lab stock
JOL2869	DH5α carrying pET28(a) + Rep	Lab stock
JOL2874	DE3 carrying pET28(a) + Cap	Lab stock
JOL2870	DE3 carrying pET28(a) + Rep	Lab stock
*E. coli* 232	F—λ—φ80 ∆(lacZYA-argF) endA1 recA1 hadR17 deoR thi-1 glnV44 gyrA96 relA1 ∆asdA4	Lab stock
JOL2881	*E. coli* 232 carrying pJHL204:Cap-Rep	This study
JOL2879	*E. coli* 232 carrying pJHL270:EuCap-Rep+ProCap	This study
Primers		
CAP P2A FW	GGGCCCATGACGTATCCAAGGAGGCGTTTC	This study
CAP-P2A33 RV	CTTCAGCAGGCTGAAGTTAGTAGCTCCGCTTCCCTTAGGGTTAAGTGGGG	This study
P2A33-ORF1 FW	CAGGCTGGAGACGTGGAGGAGAACCCTGGACCTATGCCCAGCAAGAAGAG	This study
ORF1 P2A RV	GGCGCGCCTCAGTAATTTATTTCATATGGAAATTCAGGG	This study
CAP OEP RV	CTCCAGCCTGCTTCAGCAGGCTGAAGTT	This study
ORF1 OEP FW	CCTGCTGAAGCAGGCTGGAGACGTGGAG	This study
qPCR-Cap FW	GTCTACATTTCCAGTAGTTTG	[26]
qPCR-Cap RV	CTCCCGCCATACCATAA	[26]
IFN-γ FW	TCAAGTGGCATAGATGTGGAAGAA	[27]
IFN-γ RV	TGGCTCTGCAGGATTTTCATG	[27]
TNF-α FW	CATCTTCTCAAAATTCGAGTGACAA	[27]
TNF-α RV	TGGGAGTAGACAAGGTACAACCC	[27]
IL-4 FW	ACAGGAGAAGGGACGCCAT	[27]
IL-4 RV	GAAGCCCTACAGACGAGCTCA	[27]
β-actin FW	AGAGGGAAATCGTGCGTGAC	[27]
β-actin RV	CAATAGTGATGACCTGGCCGT	[27]

## Data Availability

All data generated or analyzed during this study are included in this article.
